# Longitudinal trajectories of physical fitness in Chinese college students: a multi-group latent growth curve analysis

**DOI:** 10.3389/fpubh.2026.1917457

**Published:** 2026-08-26

**Authors:** Mei Jiang, Zhou He

**Affiliations:** 1 Sports Institute, Chengdu University of Technology, Chengdu, Sichuan, China; 2 School of Mathematics, Southwest Jiaotong University, Chengdu, Sichuan, China; 3 Sichuan Provincial Department of Education, Sichuan Province Big Data Research and Joint Application Technology Center of Student Health, Chengdu, Sichuan, China

**Keywords:** body mass index, fat but fit paradox, latent growth curve model, longitudinal trajectories, physical fitness

## Abstract

**Background:**

Physical fitness is widely regarded as an important indicator of overall health and functional capacity. However, cross-sectional studies consistently report declining fitness levels and rising overweight/obesity among college students. Longitudinal research tracking individual developmental trajectories remains scarce, and the “fat but fit paradox” is underexplored in this population. This study aimed to model 4-year trajectories of physical fitness indicators (PFI) across body mass index (BMI) categories, and to test the existence of the “fat but fit paradox”.

**Methods:**

This study used de-identified, individual-level longitudinal data comprising four annual physical fitness assessments from 183,075 Han Chinese college students across 140 universities in Chengdu, China. MG-LGCMs were constructed to examine developmental trajectories of physical fitness indicators, with baseline BMI category as the grouping variable and gender as a covariate.

**Results:**

Results revealed significant heterogeneity across BMI categories. The normal-weight group exhibited the highest baseline fitness yet showed non-significant linear growth, whereas the obese group, despite the lowest baseline, demonstrated the fastest improvement. For gravity-dependent indicators–endurance running, pull-ups (males), and 1-minute sit-ups (females)–overweight and obese groups initially displayed a disadvantage but subsequently improved significantly faster. Absolute forced vital capacity followed a “fat but fit paradox,” with higher-BMI groups maintaining superior score across 4 years. Flexibility consistently favored the normal-weight group. Gender significantly moderated both initial status and rate of change: males showed steeper acceleration across most motor domains, while females maintained baseline superiority in flexibility.

**Conclusion:**

These findings challenge uniform weight-centric assumptions about physical fitness. They highlight the need for trajectory-informed, intersectional intervention strategies in collegiate physical education and health promotion.

## 1 Introduction

Physical fitness has received much attention due to its reliability as a measure of an individual's ability to engage in physical activities and exercise, as well as an indicator of overall health (1). However, descriptive trend studies consistently indicate a decline in physical fitness among college students over recent years, characterized by reduced overall physical fitness levels (2–4), particularly in cardiorespiratory endurance, alongside a marked increase in overweight and obesity prevalence and elevated cardiovascular risk indicators such as blood pressure (2, 5), this phenomenon is closely linked to insufficient physical activity and the rise of unhealthy lifestyles (5). However, the majority of extant studies are cross-sectional, which complicates the distinction between within-person and between-person variations.

A recent longitudinal study found that between 2019 and 2022, the rate of overweight and obesity among college students continued to rise, while lower-body power, flexibility, and cardiorespiratory endurance declined at an accelerating rate. Conversely, female students exhibited annual enhancements in speed and strength (6). However, the study did not model individual trajectories over time and similarly ignored individual differences. A recent longitudinal study that employed multilevel latent growth curve modeling discovered that there were significant gender differences in the physical fitness of ethnic minority college students in China from 2015 to 2019. While their boyd mass index (BMI) remained at an optimal level during the 4-year period, their muscle strength and endurance were the weakest and showed no improvement over the 4 years, at the same time, students who exhibited a higher initial BMI demonstrated poorer outcomes in terms of flexibility, muscle strength, and endurance (7). However, the study focused only on ethnic minority groups, and the BMI categories were based on an indirect percentile-based scoring system developed for a population of Chinese students, making it difficult to extend the findings to other groups.

Furthermore, a recent study found that individuals with good physical fitness may have a lower risk of cardiometabolic diseases, even if they are overweight or obese (8). This finding is consistent with Brand's observation that optimal cardiorespiratory fitness can mitigate the adverse effects of excessive fat accumulation (9). Keating et al. (10) found among Chinese college students that the GPA trajectories of overweight or obese students actually increased more rapidly. These results finds some concurrence with the “fat but fit paradox” (11), yet extant studies have addressed this issue in only a relatively limited capacity.

Therefore, the purpose of this study is to track changes in physical fitness and its components over a 4-year period among college students, examining the existence of the so-called “fat but fit paradox” among overweight and obese individuals. This study employs multi-group latent growth curve model (MG-LGCM) to establish a framework for group-based longitudinal analysis, with the aim of revealing the heterogeneous characteristics of physical fitness changes and providing a basis for understanding the divergent pathways of physical health development.

## 2 Methods

### 2.1 Data description and sources

The data were obtained from the database of the Sichuan Province Big Data Research and Joint Application Technology Center of Student Health. The dataset comprises physical fitness test results of Han Chinese undergraduate students enrolled in 140 higher education institutions located within Chengdu, Sichuan Province. The sample was restricted to a single geographic region and ethnic group in order to minimize potential confounding from regional environmental variation and ethnic-related differences in body composition and physical development. The test indicators include height, weight, forced vital capacity, 50 m dash, sit-and-reach, standing long jump, 800 m run (female), 1,000 m run (male), 1-min sit-ups(female), and pull ups (male) (12). Prior to data delivery, the center had anonymized all personal information to ensure that no data could directly or indirectly identify individual students (including but not limited to name, detailed address, or institution name). The research protocol was authorized by the Sichuan Provincial Department of Education. As this study is a secondary analysis of strictly anonymized and de-identified data, individual informed consent was waived, and the research process fully complied with the relevant provisions of the Declaration of Helsinki regarding data use.

The original dataset comprised 185,641 longitudinal records spanning from the students' freshman year (2020) to their senior year (2023). To establish a robust longitudinal data structure and maximize data retention, a rigorous three-step data cleaning strategy was implemented. First, based on the item-specific logical check value tables from the National Student Physical Fitness and Health Survey organized by the Ministry of Education, records with missing or biologically implausible baseline BMI values were excluded, leaving 185,520 records. This initial step was indispensable, as a valid baseline grouping variable is a mathematical prerequisite for MG-LGCM. Second, to ensure the substantive meaningfulness of the trajectory modeling across all dimensions, individuals who completely lacked data across all 4 years for any single core physical fitness test were excluded as “invalid tracking” cases. Following this step, 183,075 longitudinal records were retained.

After deriving the final analytical sample (*N* = 183,075), a measurement-level data cleaning process was applied to address intermittent measurement errors. Boxplot methods were utilized to detect extreme outliers for each test at each time point. To avoid the attrition bias inherently introduced by traditional listwise deletion, the detection of extreme outliers did not result in the exclusion of the entire participant record; instead, only these specific anomalous values were recoded as missing (NA). Consequently, the final analytical panel comprised 183,075 unique individuals.

All statistical analyses and modeling were conducted using the R (version 4.2.1; R Foundation for Statistical Computing, Vienna, Austria), utilizing the packages lavaan (version 0.6.21, Ghent University, Ghent, Belgium), ggplot2 (version 3.5.0, Posit Software, PBC, Boston, MA, USA), dplyr (version 1.1.4, Posit Software, PBC, Boston, MA, USA), and semTools (version 0.5.7, CRAN / Terrence D. Jorgensen et al.).

### 2.2 Grouping design

In this study, BMI was classified according to the National Student Physical Health Standard (2014 Revision) (12), as shown in Table 1. Using the 2020 BMI data as the baseline, the 183,075 students were grouped by BMI category (see Table 2). The sample sizes of all groups met the minimum sample size requirement for statistical analysis, on this basis, descriptive statistical analysis was performed for each physical fitness test item by group.

**Table 1:** BMI classification standard.

Gender	Underweight	Normal weight	Overweight	Obese
Male	< 17.9	17.9–23.9	24.0–27.9	>27.9
Female	< 17.2	17.2–23.9	24.0–27.9	>27.9

**Table 2:** Sample distribution by gender and BMI classification.

Gender	Underweight	Normal weight	Overweight	Obese	Total
Male	7,720 (9.4%)	55,857 (68.3%)	12,884 (15.8%)	5,282 (6.5%)	81,743
Female	6,694 (6.6%)	83,571 (82.5%)	8,531 (8.4%)	2,536 (2.5%)	101,332
Total	14,414	139,428	21,415	7,818	183,075

### 2.3 Physical fitness indicator (PFI)

To investigate the overall trend of physical fitness levels across the four-year university period, we used the physical fitness indicator (PFI) (13, 14) to represent physical fitness level. The PFI is calculated as the sum of the Z-scores of each physical fitness test indicator. Notably, lower completion times in the 50 m dash, 1,000 m run (male), and 800 m run (female) indicate better physical fitness; therefore, the *Z*-scores of these three timed items were multiplied by –1 for reverse processing, so that higher scores on all items uniformly represent better physical fitness. A higher PFI value indicates a better overall physical fitness level.

### 2.4 Multi-group latent growth curve model (MG-LGCM)

A multi-group latent growth curve model (MG-LGCM) was employed to examine trajectories of physical fitness change. After group classification, a linear latent growth model was specified using annual physical fitness test scores collected across four waves as repeated outcome measures. The intercept factor captured initial physical fitness levels, while the slope factor reflected the linear annual rate of change. All models were fitted in the R package lavaan using robust maximum likelihood (MLR) estimation with full information maximum likelihood (FIML) to address non-normality and missing at random, thereby yielding robust parameter estimates.

#### 2.4.1 Unconditional linear growth model

An unconditional linear latent growth model was first specified using the entire sample. The model included two latent growth factors: an intercept factor (i) and a linear slope factor(s). Loadings from the intercept factor to the four measurement occasions (2020, 2021, 2022, and 2023) were fixed at 1, representing the baseline level, while loadings from the slope factor were fixed at 0, 1, 2, and 3, respectively, assuming linear change across equally spaced time points. The model can be expressed as:


$$\begin{array}{c} Y_{ti}=\eta_{0i}+\lambda_{t}\eta_{1i}+\varepsilon_{ti}\\ \eta_{0i}=\alpha_{0}+\zeta_{0i},\;\eta_{1i}=\alpha_{1}+\zeta_{1i}, \end{array}$$

where η_0*i*_ and η_1*i*_ are the individual-level intercept and linear slope, respectively; α_0_ and α_1_ are their corresponding means; and ζ_0*i*_ and η_1*i*_ are the individual deviations from these means, with variances and covariances freely estimated. After this model demonstrated good fit in the total sample, it was subsequently tested separately in each of the four weight groups to confirm that the linear functional form was acceptable for all groups.

#### 2.4.2 Multiple-group baseline model and omnibus test of mean differences

After configural invariance was established, a multiple-group baseline model (*M*_0_) was specified. In this model, all parameters were freely estimated, serving as the reference for all subsequent comparisons.

To rigorously evaluate overall between-group differences in the growth parameters, a parameter equality constraint method was employed. Three nested models were specified and compared against the baseline model (*M*_0_) to test the global null hypotheses (*H*_0_) of parameter invariance:

- Model 1 (equal intercept means): Constrained intercept means to be equal across groups (*H*_0*i*_:α_0, *normal*_ = α_0, *overweight*_ = α_0, *underweight*_ = α_0, *obese*_).
- Model 2 (equal slope means): Constrained slope means to be equal across groups (*H*_0*s*_:α_1, *normal*_ = α_1, *overweight*_ = α_1, *underweight*_ = α_1, *obese*_).
- Model 3 (equal intercept and slope means): Imposed both sets of constraints simultaneously.

Because robust maximum likelihood (MLR) estimation was utilized, the conventional chi-square difference test is invalid. Therefore, nested model comparisons were formally conducted using the Satorra-Bentler scaled chi-square difference test ($\Delta \chi_{SB}^{2}$), which corrects the test statistic using scaling factors provided by the MLR estimator (15). A significant $\Delta \chi_{SB}^{2}$ indicates the rejection of *H*_0_, thereby confirming global heterogeneity in the respective growth parameters across the four BMI groups.

#### 2.4.3 Post-hoc pairwise comparisons and statistical significance

Following a significant global test result, specific *post-hoc* comparisons were conducted to identify where the exact differences lay. These comparisons were explicitly tested by imposing linear equality constraints between any two specific BMI groups (e.g., *H*_0_:α_*g*1_−α_*g*2_ = 0) within the baseline model framework.

These local constraints were evaluated using multivariate Wald tests, which compute a test statistic by evaluating the constraints against the parameters' asymptotic covariance matrix. Statistical significance for all omnibus and *post-hoc* tests in this study was established at a two-tailed α-level of 0.05.

### 2.5 Handling of missing data and justification for FIML

To fully harness the analytical power of the longitudinal design, the final panel (*N* = 183,075) included all participants who had at least one valid observation in each core physical fitness dimension across the four-year study period. All unobserved test scores due to intermittent absenteeism or localized outlier exclusion were objectively coded as missing.

Prior to the application of Full Information Maximum Likelihood (FIML) estimation, a rigorous attrition analysis was conducted to empirically test the Missing At Random (MAR) assumption. A binary logistic regression model was employed to predict the likelihood of intermittent missingness across the tracking period using baseline covariates. The results clearly demonstrated that the missingness was not Completely At Random (MCAR). Rather, the propensity for intermittent missingness was significantly predicted by observed baseline characteristics: having a higher baseline BMI (*p* = 0.025) and possessing a higher initial absolute forced vital capacity (*p* < 0.001) were significantly associated with an increased probability of missing subsequent tests, whereas gender did not show a statistically significant effect in this attrition model (*p* = 0.210).

Because the probability of missingness can be systematically explained by these baseline covariates, and these variables were explicitly incorporated into the multi-group modeling framework, the MAR assumption was robustly satisfied (16). Consequently, the FIML algorithm was strictly implemented via the lavaan package to compute unbiased and efficient parameter estimates directly from the incomplete longitudinal trajectories, thereby effectively circumventing the estimation bias associated with listwise deletion.

## 3 Results

### 3.1 Descriptive statistics

**Figure 1:**
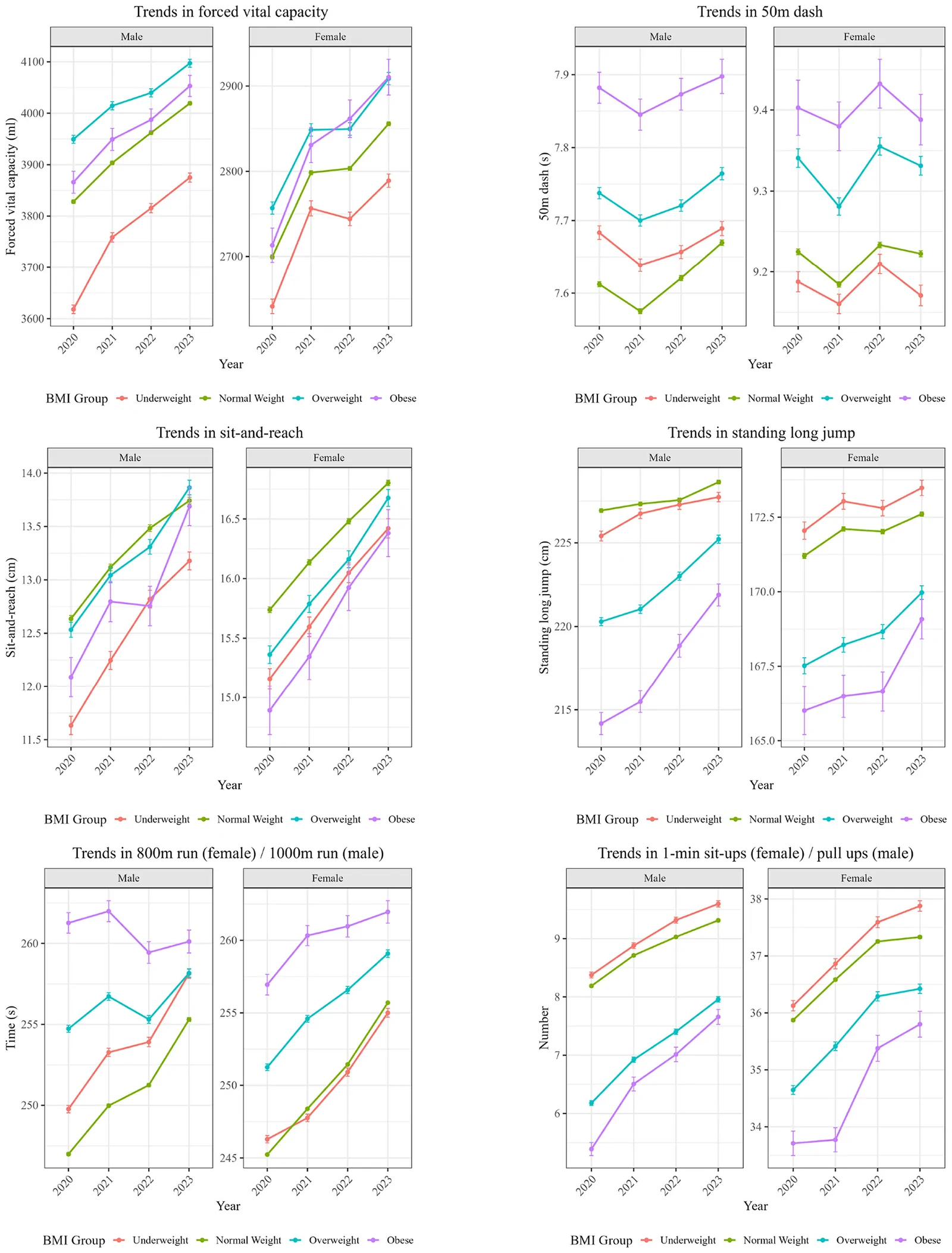
Trends in physical fitness performance by BMI group.

Figure 1 presents the line graphs of changes in college students' physical fitness test scores for each item from 2020 to 2023. Overall, during this period, the scores for forced vital capacity, standing long jump, sit-and-reach, 1-min sit-ups (female), and pull ups (male) showed an improving trend, whereas the performance in the 50 m dash, 800 m run (female), and 1,000 m run (male) declined. This indicates that students' flexibility and strength improved during the college years, while their speed and endurance declined.

### 3.2 Trends in physical fitness indicator

#### 3.2.1 Temporal trends in PFI

**Figure 2:**
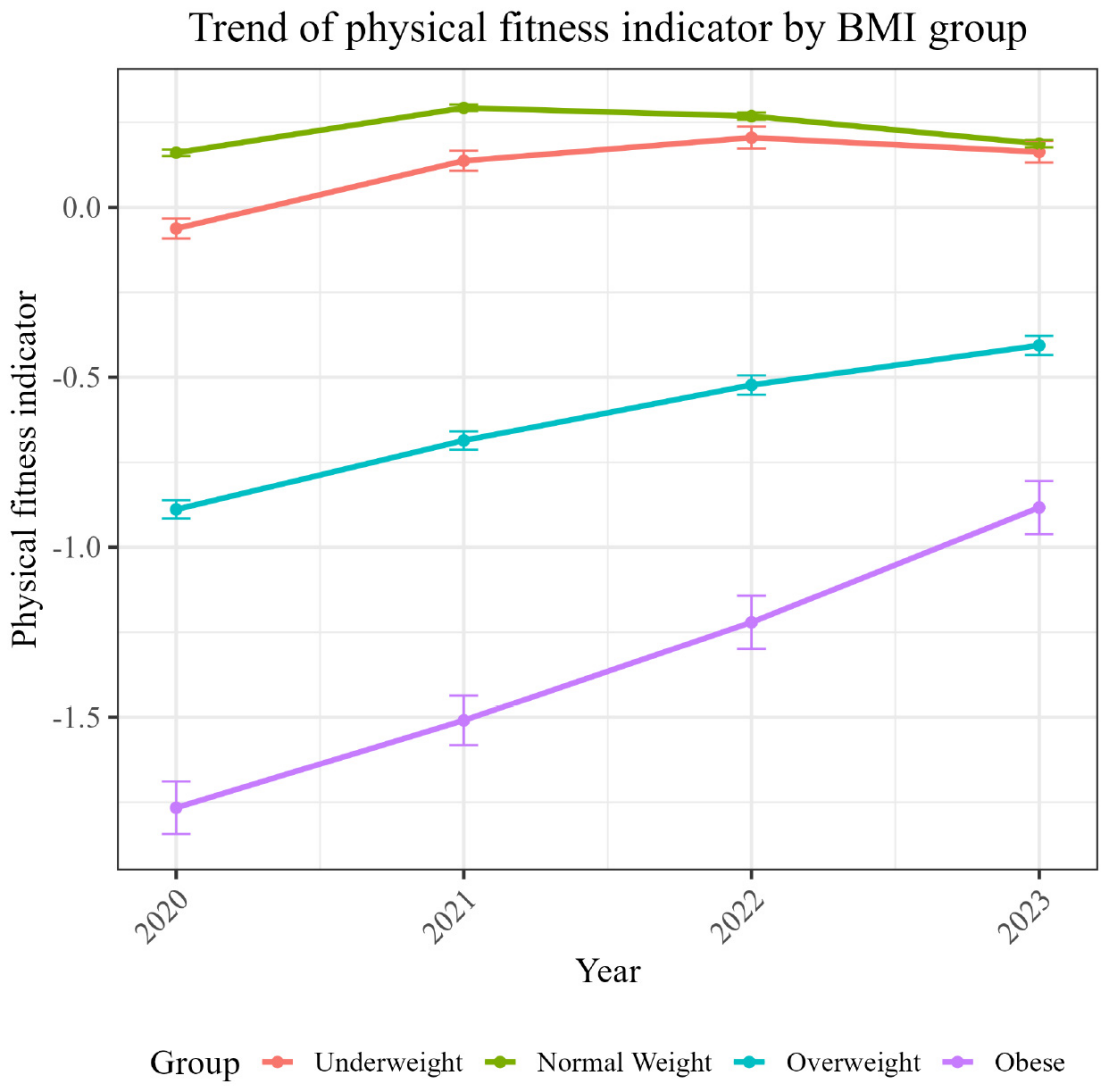
Temporal trends of PFI across BMI groups from 2020 to 2023.

The temporal trends of physical fitness index (PFI) across BMI groups from 2020 to 2023 are presented in Figure 2. The normal weight group consistently exhibited the highest PFI at all time points, while the obese group had the lowest. All groups showed an overall upward trend in PFI over the study period, although the magnitude of increase differed. The normal weight group displayed the flattest trajectory, with a tendency to decline after 2021. In contrast, the overweight and obese groups demonstrated more pronounced improvements, whereas the underweight group occupied an intermediate position.

#### 3.2.2 Overall linear latent growth model

First, a linear latent growth model was estimated for the entire sample (without grouping) to examine the overall developmental trajectory of PFI. The model fit indices were as follows: $\chi_{\left(5\right)}^{2}=9034.24$, *p* < 0.001; CFI = 0.942; TLI = 0.930; RMSEA = 0.130, 90% CI [0.128, 0.132]; SRMR = 0.063. Although the chi-square test was significant and the RMSEA was relatively high (likely attributable to the very large sample size), both CFI and TLI exceeded 0.90 and SRMR was below 0.08, indicating that the model was acceptable.

Parameter estimates revealed that the mean latent intercept (*i*) was 0.057 (SE = 0.009, *p* < 0.001) and the mean latent slope (*s*) was 0.032 (SE = 0.003, *p* < 0.001). This suggests that, on average, students started with a positive PFI level at baseline and showed a small linear increase over the study period. Both the intercept variance (5.582, *p* < 0.001) and slope variance (0.540, *p* < 0.001) were significant, indicating substantial inter-individual heterogeneity in initial PFI and in the rate of change. The intercept-slope covariance was –0.656 (*p* < 0.001), implying that individuals with higher initial PFI tended to exhibit slower growth over time.

Standardized factor loadings showed that the loadings of the four time-point PFI measures on the intercept factor ranged from 0.74 to 0.85, while loadings on the slope factor increased from 0 (fixed) to 0.688, supporting the appropriateness of the linear growth specification.

#### 3.2.3 Multi-group latent growth model and cross-group comparison

A multigroup analysis was conducted across the four BMI groups (normal weight, overweight, underweight, and obese) to examine between-group differences in the developmental trajectories of PFI. The baseline model, in which all parameters were freely estimated, yielded the following fit indices: $\chi_{\left(20\right)}^{2}=9068.89$, *p* < 0.001; CFI = 0.940; TLI = 0.929; RMSEA = 0.130, 90% CI [0.128, 0.132]; SRMR = 0.064, indicating acceptable overall fit.

The latent mean estimates for the four groups are presented in Table 3. The intercept mean (*i*) reflects the initial PFI level in 2020. The normal weight group had the highest intercept (0.222, *p* < 0.001), followed by the underweight group (–0.003, *p* = 0.927, not significantly different from zero). The overweight group was significantly lower than the normal weight group (–0.866, *p* < 0.001), and the obese group had the lowest intercept (–1.792, *p* < 0.001). The slope mean (*s*) represents the linear rate of change across the four years. All groups exhibited positive slope estimates. However, the slope of the normal weight group was not significant (0.006, *p* = 0.132), whereas the underweight group (0.075, *p* < 0.001), overweight group (0.161, *p* < 0.001), and obese group (0.296, *p* < 0.001) all showed significant positive growth. The obese group demonstrated the fastest rate of increase.

**Table 3:** Unstandardized parameter estimates from the multi-group latent growth model.

Parameter	Normal weight	Overweight	Underweight	Obese
Latent means				
Intercept *i*	0.222(0.010)^***^	−0.866(0.026)^***^	−0.003(0.029)	−1.792(0.073)^***^
Slope *s*	0.006(0.004)	0.161(0.011)^***^	0.075(0.012)^***^	0.296(0.030)^***^
Latent variances				
*i* variance	5.457(0.042)^***^	5.423(0.120)^***^	5.079(0.127)^***^	5.344(0.342)^***^
*s* variance	0.527(0.009)^***^	0.547(0.028)^***^	0.623(0.029)^***^	0.570(0.080)^***^
Latent covariance				
*i* with *s*	−0.615(0.016)^***^	−0.663(0.045)^***^	−0.770(0.049)^***^	−0.646(0.125)^***^

Standard errors are shown in parentheses. The symbols *, **, and *** indicate statistical significance at the 5%, 1%, and 0.1% levels, respectively.

To test the statistical significance of between-group differences, we imposed equality constraints on the intercept means, the slope means, and both sets of means across groups, respectively, and compared each constrained model against the baseline model. Satorra-Bentler scaled chi-square difference tests showed that constraining the intercept means to be equal significantly worsened model fit, $\Delta \chi_{\left(3\right)}^{2}=1685.1$, *p* < 0.001; constraining the slope means to be equal also led to significantly worse fit, $\Delta \chi_{\left(3\right)}^{2}=281.29$, *p* < 0.001; and constraining both intercept and slope means to be equal similarly resulted in significantly worse fit, $\Delta \chi_{\left(6\right)}^{2}=1971.1$, *p* < 0.001. These results indicate that the four BMI groups differ significantly in both the initial level and the rate of change of PFI.

Pairwise comparisons were further examined using Wald tests. All six pairwise comparisons were significant (*p* < 0.001), with detailed statistics reported in Table 4. For the intercept means, the normal weight group was significantly higher than the other three groups, the underweight group was significantly higher than the overweight and obese groups, and the overweight group was significantly higher than the obese group. For the slope means, the obese group exhibited a significantly faster rate of increase than the overweight group (χ^2^ = 45.74, *p* < 0.001), the overweight group significantly faster than the underweight group (χ^2^ = 29.27, *p* < 0.001), and the underweight group significantly faster than the normal weight group (χ^2^ = 192.13, *p* < 0.001). This pattern reveals a stepwise distribution in which higher BMI categories are associated with greater increases in PFI over time.

**Table 4:** Wald test results for pairwise comparisons of latent means.

Comparison	Intercept *i* χ^2^(1)	*p*	Slope *s* χ^2^(1)	*p*
Underweight vs. normal weight	1,542.42	< 0.001	192.13	< 0.001
Underweight vs. overweight	54.51	< 0.001	29.27	< 0.001
Underweight vs. obese	741.11	< 0.001	89.89	< 0.001
Normal weight vs. overweight	492.20	< 0.001	28.80	< 0.001
Normal weight vs. obese	141.46	< 0.001	17.62	< 0.001
Overweight vs. obese	514.58	< 0.001	45.74	< 0.001

Notably, although the obese group had the lowest initial PFI (–1.792), its linear slope reached 0.296 (*p* < 0.001), which was significantly faster than that of the normal weight group (0.006, *p* = 0.132) and the other groups, indicating that obese students experienced the greatest improvement in physical fitness. This pattern is consistent with certain features of the “fat but fit paradox,” whereby individuals with higher body weight may exhibit more positive adaptive changes in health behaviors or physical fitness development (17–19).

#### 3.2.4 The influence of gender on the developmental trajectories of PFI

A gender dummy variable was added to the baseline model, allowing the effects of gender on the latent intercept and slope to be freely estimated across groups. The model yielded the following fit indices: $\chi_{\left(28\right)}^{2}=9098.76$, *p* < 0.001; CFI = 0.940; TLI = 0.915; RMSEA = 0.110, 90% CI [0.108, 0.112]; SRMR = 0.053. Parameter estimates for the gender effects are presented in Table 5.

**Table 5:** Regression coefficients of gender on the latent intercept and slope.

BMI group	Intercept *i* ←female *b* (SE)	*p*	Slope *s* ←female *b* (SE)	*p*
Normal weight	−0.193(0.020)	< 0.001	−0.026(0.008)	0.001
Overweight	0.257(0.052)	< 0.001	−0.140(0.021)	< 0.001
Underweight	0.224(0.058)	< 0.001	−0.049(0.024)	0.046
Obese	0.545(0.148)	< 0.001	−0.189(0.061)	0.002

In the normal weight group, females had a significantly lower intercept than males (*b* = −0.193, *p* < 0.001) and also a significantly lower slope (*b* = −0.026, *p* = 0.001), indicating that normal weight girls started with lower initial PFI and improved at a slower rate. In the overweight (*b*_*i*_ = 0.257, *p* < 0.001), underweight (*b*_*i*_ = 0.224, *p* < 0.001), and obese (*b*_*i*_ = 0.545, *p* < 0.001) groups, females had significantly higher initial PFI than males. However, in these three groups, females still exhibited significantly lower slopes than males (overweight: *b*_*s*_ = −0.140, *p* < 0.001; underweight: *b*_*s*_ = −0.049, *p* = 0.046; obese: *b*_*s*_ = −0.189, *p* = 0.002), suggesting that despite their initial advantage, their rate of PFI increase remained slower than that of males in the same group.

**Figure 3:**
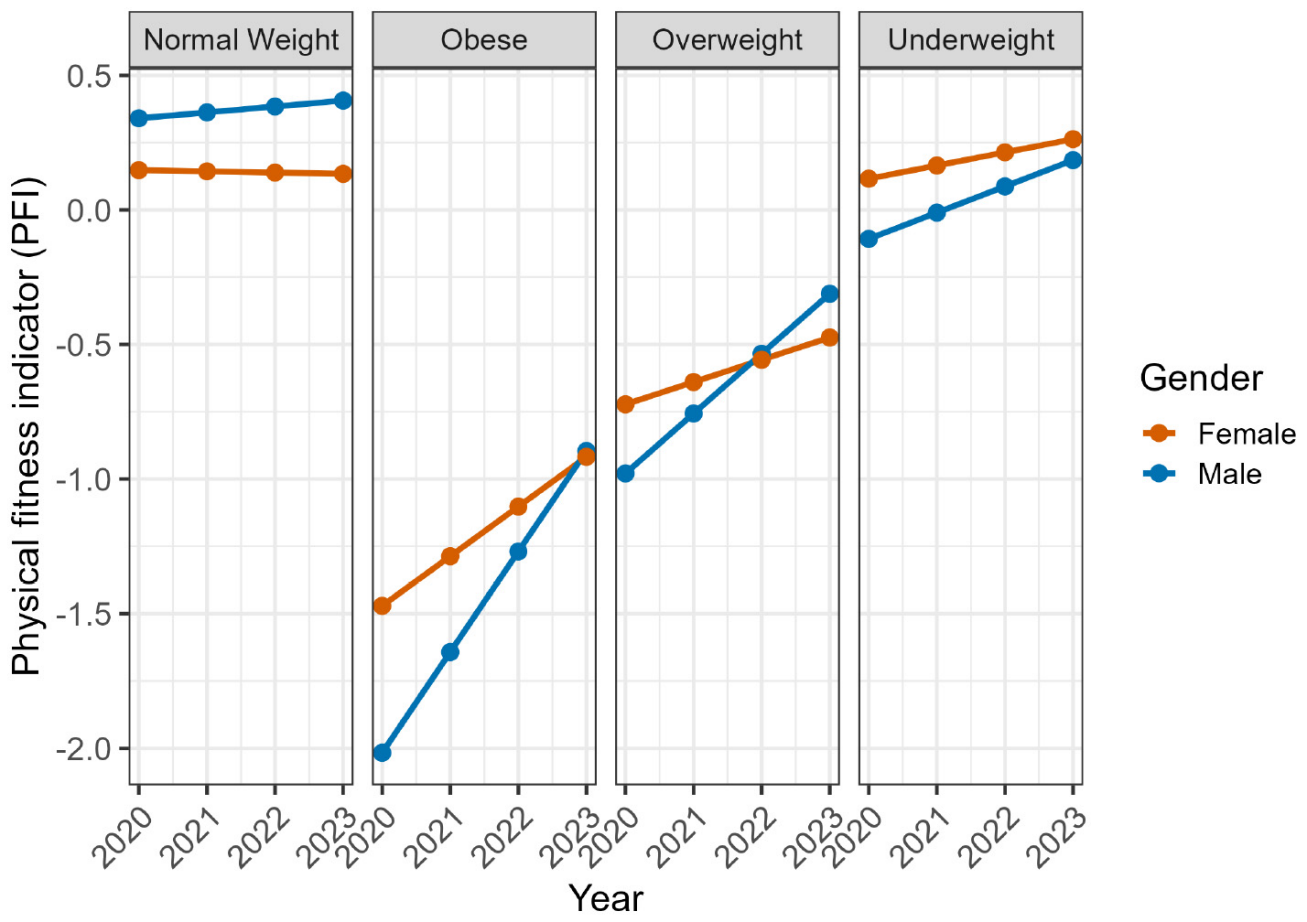
Developmental trajectories of PFI by BMI group and gender.

Tests constraining the gender effects to be equal across groups showed that the gender effects on the intercept were not equal across groups [$\Delta \chi_{\left(3\right)}^{2}=121.72$, *p* < 0.001], nor were the gender effects on the slope [$\Delta \chi_{\left(3\right)}^{2}=31.78$, *p* < 0.001]; constraining both sets of effects to be equal was also significant [$\Delta \chi_{\left(6\right)}^{2}=134.73$, *p* < 0.001]. These findings indicate that BMI group moderates the relationship between gender and both the initial level and the developmental trajectory of PFI. The gender-specific predicted trajectories generated from the estimated parameters (Figure 3) visually illustrate this pattern: in the normal weight group, the trajectories for males and females were relatively close with high starting points; in the other three groups, females had higher starting points but flatter slopes, whereas males had relatively lower starting points but steeper increases.

### 3.3 Analysis of between-group and gender differences in the developmental trajectories of each physical fitness test item

To further investigate the specific influence of BMI on students' physical fitness development, this study employed a multi-group latent growth model to analyze the developmental trajectories of six physical fitness test items separately. Based on the biomechanical and physiological characteristics of each item, they were categorized into three types: gravity-dependent indicators (50 m dash, standing long jump, pull ups (male)/1-min sit-ups(female), and 1,000 m run (male)/800 m run (female)), an absolute functional indicator (forced vital capacity), and a flexibility indicator (sit-and-reach). The results of the measurement invariance tests for all models are summarized in Table 6. The findings showed that the configural invariance model for each indicator achieved acceptable fit (CFI > 0.92, TLI > 0.90, SRMR < 0.08). Although the RMSEA was somewhat elevated in a few models, this was considered acceptable given the extremely large sample size and the simplified specification of linear trajectories (20, 21). Thus, the prerequisite for cross-group comparison of latent means was satisfied.

**Table 6:** Fit indices and measurement invariance tests of multi-group models for each physical fitness test item.

Indicator	CFI	TLI	RMSEA [90% CI]	SRMR	Strict invariance *Δχ*^2^ (Δdf)
Gravity-dependent indicators					
50 m dash	0.985	0.982	0.093 [0.091, 0.095]	0.033	32.05(12)^**^
Standing long jump	0.990	0.988	0.093 [0.091, 0.095]	0.022	44.94(12)^***^
Pull ups (male)	0.946	0.936	0.125 [0.121, 0.128]	0.063	20.75(12)
1-min sit-ups (female)	0.926	0.912	0.122 [0.120, 0.125]	0.056	27.06(12)^*^
1,000 m run (male)	0.892	0.870	0.151 [0.148, 0.155]	0.075	108.26(12)^***^
800 m run (female)	0.922	0.906	0.128 [0.125, 0.131]	0.066	44.06(12)^***^
Absolute functional indicator					
Forced vital capacity	0.982	0.978	0.103 [0.101, 0.106]	0.033	43.65(12)^***^
Relative functional indicator					
FWI	0.948	0.937	0.132 [0.130, 0.134]	0.057	853.64(12)^***^
Flexibility indicator					
Sit-and-reach	0.963	0.955	0.097 [0.094, 0.099]	0.045	46.54(12)^***^

^*^p < 0.05, ^**^p < 0.01, ^***^p < 0.001.

#### 3.3.1 Gravity-dependent indicators

**Figure 4:**
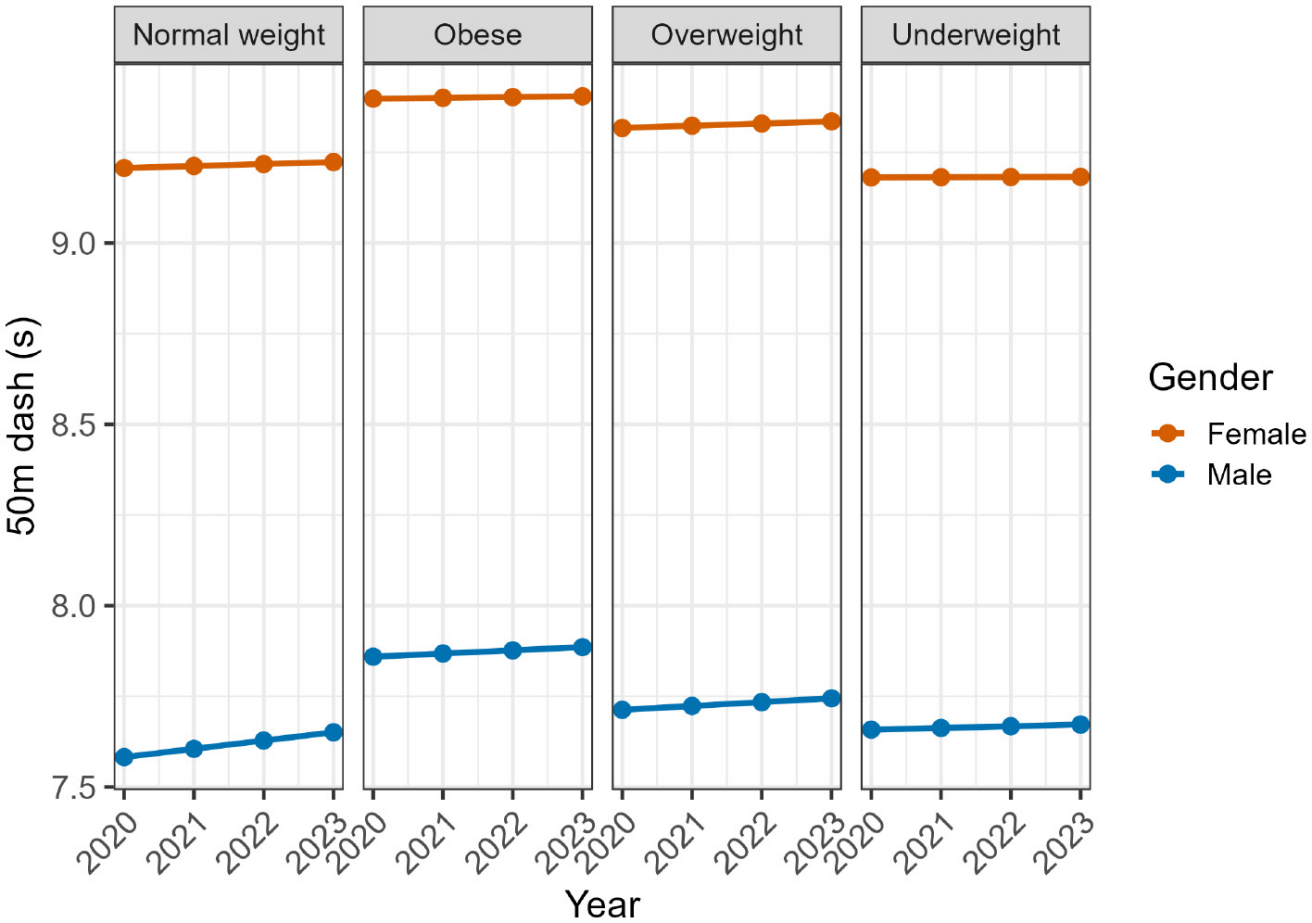
Predicted 50 m dash trajectories by BMI group and gender.

**Figure 5:**
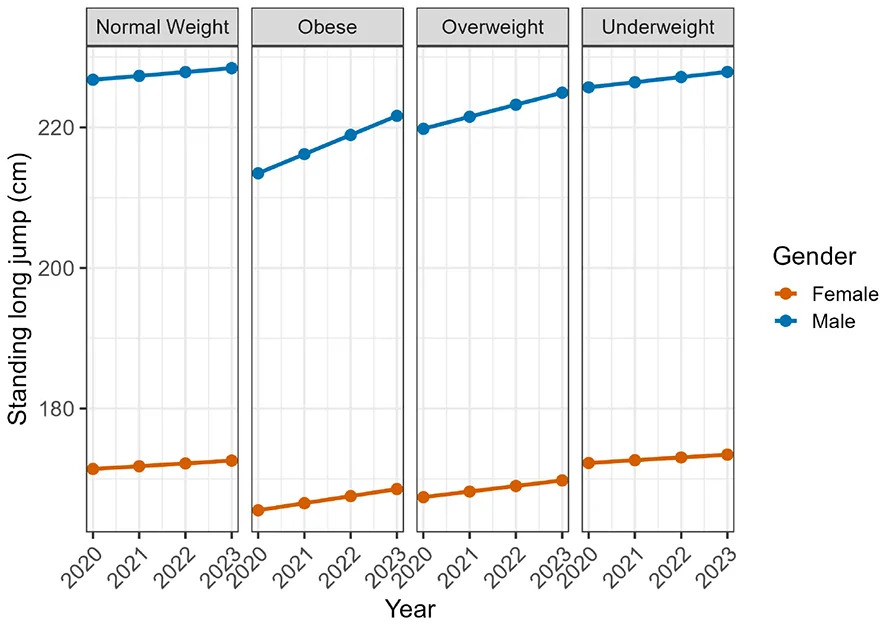
Predicted trajectories of standing long jump by BMI group and gender.

**Figure 6:**
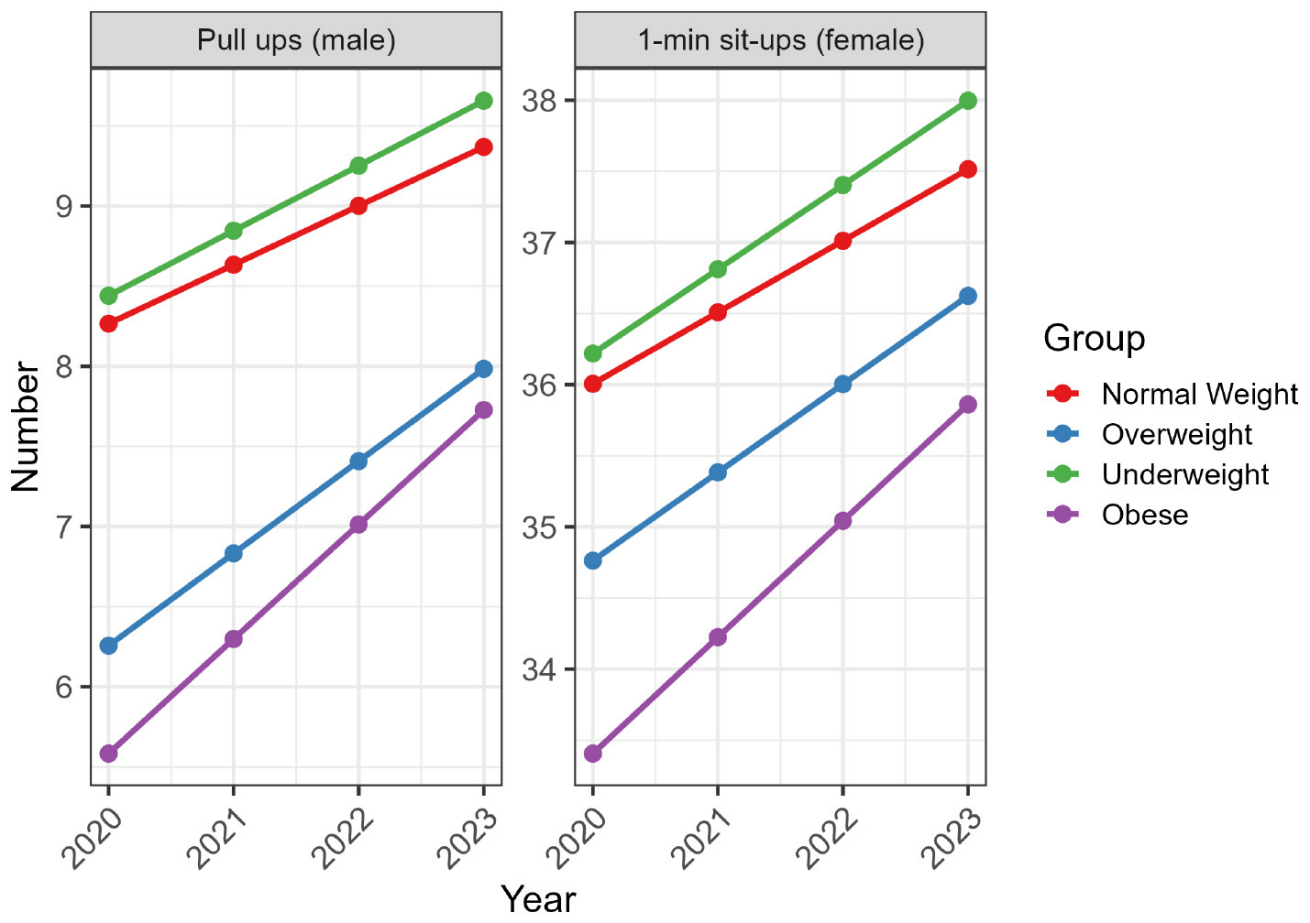
Predicted trajectories of pull ups (male)/1-min sit-ups (female) by BMI group.

**Figure 7:**
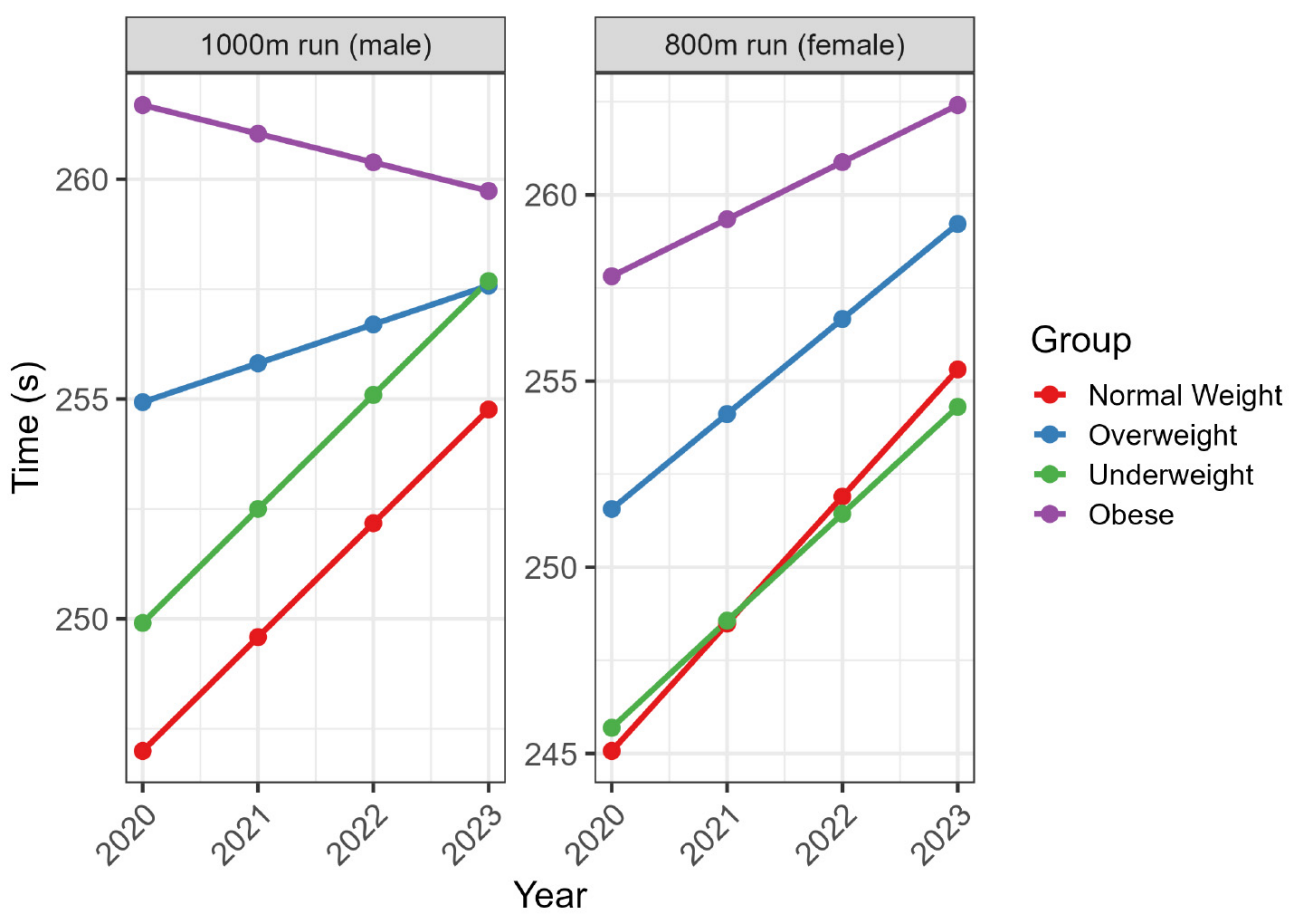
Predicted trajectories of 1,000 m run (male)/800 m run (female) by BMI group.

Gravity-dependent indicators require individuals to overcome or utilize their own body weight to execute movements, and performance on these items (see Figures 4–7) is closely linked to body composition and absolute strength. Overall, indicators in this category consistently exhibit a baseline disadvantage for overweight and obese students, yet they also display a faster rate of improvement in the standing long jump and strength-endurance items, giving rise to a discernible catch-up effect.

##### 3.3.1.1 Between-group differences in trajectories across BMI groups

In the endurance running (1,000 m run (male)/800 m run (female)) and body muscle strength (pull ups (male)/1-min sit-ups(female)), which require overcoming one's own body weight, baseline performance showed a clear stepwise pattern: the obese group performed worst, followed by the overweight group, and the normal weight and underweight groups performed best, with all between-group differences reaching statistical significance, reflecting a typical disadvantage associated with excess body weight (*p* < 0.001). For example, the initial 1,000 m run (male) time for obese boys (261.69 s) was nearly 15 s slower than that of normal weight boys (247.00 s). In terms of the rate of change, however, a contrasting catch-up pattern was observed: the overweight and obese groups showed significantly less decline (in endurance running) or greater improvement (in pull ups (male)/1-min sit-ups(female)) compared with the normal weight group. Notably, in boys' pull ups, the obese group exhibited an average annual increase of 0.72 repetitions, significantly higher than the 0.37 repetitions recorded for the normal weight group (*p* < 0.001). This dynamic process is visually captured in Figures 6, 7: although all BMI groups displayed an upward trend across the years, the slopes for the overweight and obese groups were steeper, so that the between-group gaps did not widen significantly over the four-year period and even narrowed in the strength items.

The 50 m dash and standing long jump did not exhibit the disadvantage associated with excess body weight. As shown in Figures 4, 5, for both sexes, the normal weight and underweight groups had significantly faster 50 m dash times than the overweight and obese groups. Similarly, in the standing long jump, the initial scores of the normal weight group were also significantly higher than those of the obese group.

##### 3.3.1.2 Moderating effect of gender

The effect of gender on initial levels was highly significant for all items (*p* < 0.001), with girls systematically performing worse than boys. The gender gap (reflected by the regression coefficient) was typically smallest in the underweight group and larger in the normal weight or overweight groups. For instance, in the standing long jump, the initial disadvantage of girls relative to boys was smaller in the obese group (–47.94 cm) than in the normal weight group (–55.38 cm). More importantly, the moderating effect of gender on the rate of change differed across items. In the standing long jump, girls in all BMI groups showed a significantly slower rate of improvement than boys, causing the gender gap to widen over time; this trend is clearly visible in Figure 5, where the upward trajectory for boys in the obese group is particularly steep. In contrast, for the 50 m dash and endurance running, the effect of gender on the rate of change was non-significant in most groups, with the boys' and girls' curves being nearly parallel in Figure 4. This suggests that, during the college years, changes in the gender gap for gravity-dependent indicators are largely determined by the nature of the item itself.

#### 3.3.2 Absolute functional indicator

As an absolute indicator reflecting respiratory functional potential, forced vital capacity exhibits a developmental pattern markedly distinct from that of gravity-dependent indicators.

##### 3.3.2.1 Between-group differences in trajectories across BMI groups

**Figure 8:**
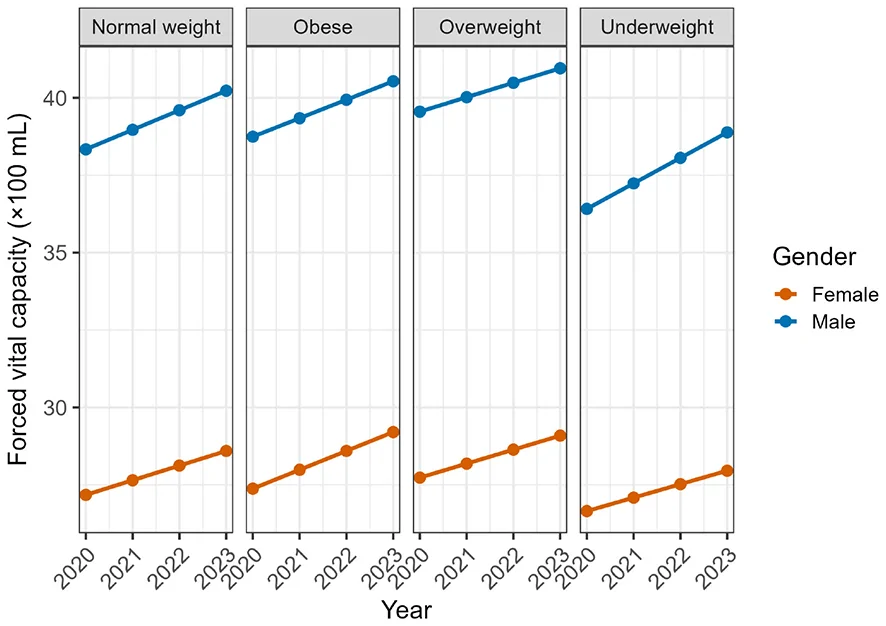
Predicted forced vital capacity trajectories by BMI group and gender.

In contrast to the “obesity disadvantage,” baseline forced vital capacity displayed a pronounced “fat but fit paradox.” The overweight (34.36) and obese (34.08) groups both had significantly higher baseline forced vital capacity than the normal weight group (31.53; *p* < 0.001). Although the average annual rate of increase for the overweight and obese groups (slope range: 0.45–0.60) was slightly lower than that of the normal weight group (0.53), their substantial initial advantage enabled their absolute forced vital capacity to remain consistently at or above the level of the normal weight group throughout the four-year college period. This paradoxical pattern is illustrated in Figure 8, where the trajectory curves of the overweight and obese groups run above those of the other groups on the vertical axis and are nearly parallel to them.

##### 3.3.2.2 Moderating effect of gender

Gender had an extremely strong effect on initial levels (*p* < 0.001), with girls systematically demonstrating lower forced vital capacity than boys. The magnitude of this gender gap varied across BMI groups, being relatively smallest in the underweight group. Regarding the rate of change, girls in the normal weight and underweight groups improved significantly more slowly than boys (*p* < 0.001), causing the gender gap in these two groups to widen over time; in the overweight and obese groups, however, the gender difference in rate of change was non-significant. These findings jointly shaped the trajectories shown in Figure 8, such that the advantages of high BMI and male gender were superimposed, while the disadvantage of girls in the underweight group was particularly pronounced.

#### 3.3.3 Relative functional indicator

To account for the confounding effect of body mass on pulmonary function, we evaluated a relative indicator: the forced vital capacity-to-weight index (FWI) (22).

##### 3.3.3.1 Between-group differences in trajectories across BMI groups

A multi-group latent growth model with an intercept and linear slope was fit to the FWI across four annual assessments. The model allowed all latent means, variances, and covariances to vary freely across the four BMI groups, yielding acceptable approximate fit (CFI = 0.948, TLI = 0.937, RMSEA = 0.132, SRMR = 0.057).

**Figure 9:**
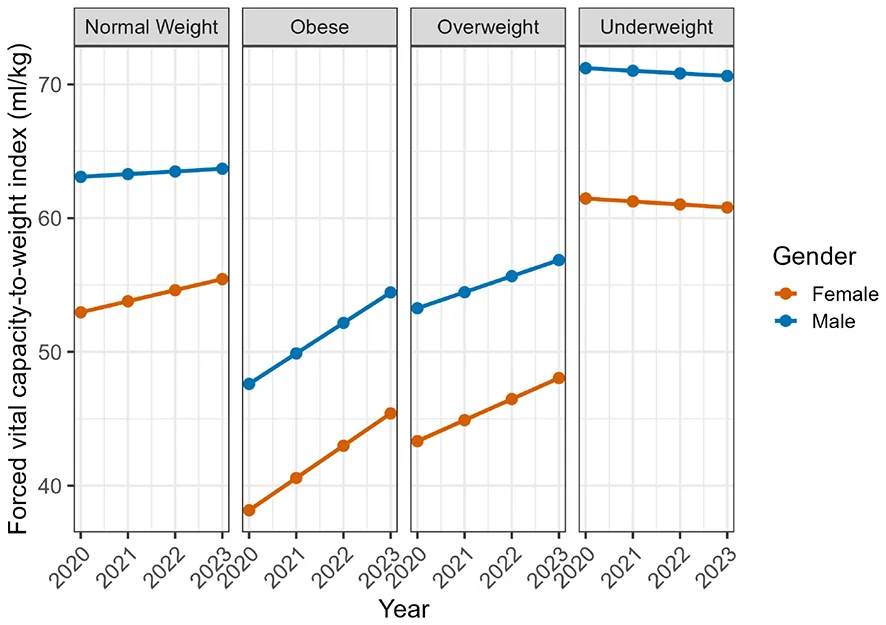
Predicted forced vital capacity-to-weight index trajectories by BMI group and gender.

In contrast to the pattern observed for absolute forced vital capacity, the baseline FWI exhibited a marked underweight advantage. The underweight group had the highest initial level (66.64), followed by the normal weight (56.92), overweight (48.95), and obese (43.85) groups. Pairwise Wald tests confirmed that all intercept means differed significantly from one another (*p* < 0.001). The rate of change also diverged sharply across groups: the obese group showed the steepest annual increase (slope = 2.31), followed by the overweight (1.35) and normal weight (0.58) groups, whereas the underweight group declined slightly over time (slope = –0.21). All slopes were significantly different from zero and from each other (*p* < 0.001). Consequently, although the obese and overweight groups started at substantially lower levels, their faster growth led to a marked convergence over the four-year period, while the underweight group's initial advantage eroded over time. These diverging trajectories are illustrated in Figure 9.

##### 3.3.3.2 Moderating effect of gender

To examine whether gender moderated the growth trajectories, the latent intercept and slope were regressed on gender, with effects freely estimated in each group. The conditional model fit the data adequately (CFI = 0.950, TLI = 0.928, RMSEA = 0.118, SRMR = 0.044).

Gender had a strong and consistent effect on initial FWI. Constraining the gender gap on the intercept to be equal across groups did not significantly worsen model fit [$\Delta \chi_{\left(3\right)}^{2}$ = 5.87, *p* = 0.118], indicating that the disadvantage of females at baseline was uniform across weight-status groups. In all four groups, females exhibited a significantly lower intercept than males, with unstandardized coefficients ranging from –9.44 to –10.13 (*p* < 0.001). The effect of gender on the rate of change, however, varied by BMI group. Imposing equality constraints on the gender-by-slope interaction significantly degraded fit [$\Delta \chi_{\left(3\right)}^{2}$ = 49.67, *p* < 0.001]. In the normal weight group, females demonstrated a significantly steeper slope than males (*b* = 0.63, *p* < 0.001), and a similar, though smaller, female advantage was observed in the overweight group (*b* = 0.37, *p* < 0.001). In contrast, the gender difference in slope was non-significant for the underweight (*b* = –0.03, *p* = 0.770) and obese (*b* = 0.13, *p* = 0.485) groups. As shown in Figure 9, these differential effects shaped the trajectories such that the gender gap narrowed over time in the normal weight and overweight groups, while remaining relatively stable in the underweight and obese groups.

#### 3.3.4 Flexibility indicator

The sit-and-reach, as a typical indicator of flexibility, exhibited a distinctive developmental pattern that aligns more closely with conventional health perceptions.

##### 3.3.4.1 Between-group differences in trajectories across BMI groups

**Figure 10:**
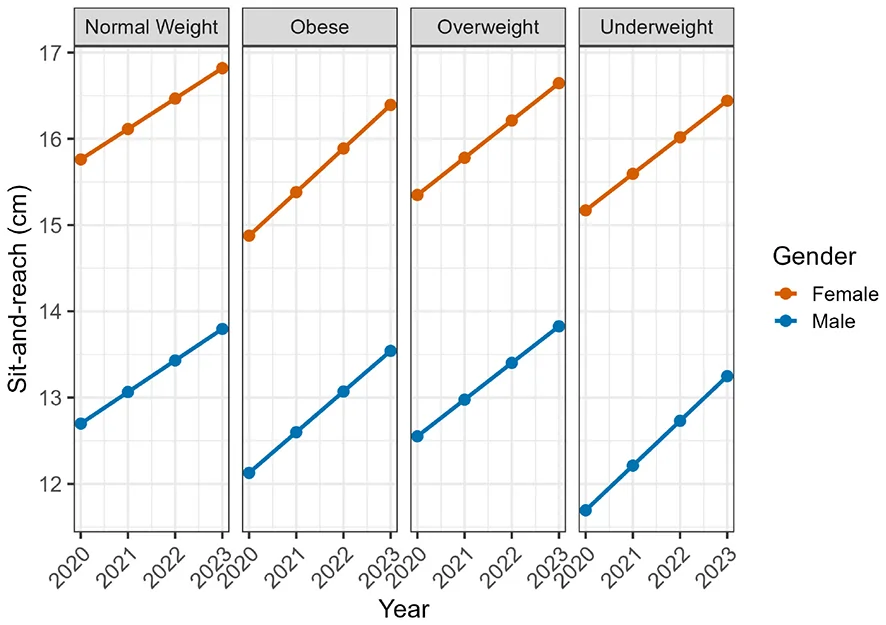
Predicted sit-and-reach trajectories by BMI group and gender.

The normal weight group demonstrated significantly better baseline flexibility (14.57 cm) and final level than all other groups, indicating a clear “normal-weight advantage.” The overweight group (13.79 cm) ranked second, whereas the underweight (13.33 cm) and obese (13.26 cm) groups performed worst, with no significant baseline difference between the latter two (*p* = 0.645). Although the underweight, overweight, and obese groups each exhibited a significantly faster average annual rate of increase than the normal weight group (*p* < 0.05), resulting in some degree of catch-up, the normal weight group's advantage persisted through the senior year. This trend is clearly visible in Figure 10: the trajectory of the normal weight group lies at the top, with the remaining groups arranged in descending order; the between-group gaps narrowed but the ranking remained unchanged.

##### 3.3.4.2 Moderating effect of gender

Gender had a significant effect on baseline flexibility, with girls outperforming boys in all BMI groups (*p* < 0.001). The magnitude of this female advantage was more pronounced in the underweight and normal weight groups. Notably, in stark contrast to the large gender difference at baseline, gender exerted virtually no significant moderating effect on the rate of change over the four-year period, indicating that changes in flexibility during the college years were primarily driven by BMI group rather than gender. The generally parallel trajectories of boys and girls in Figure 10 provide direct visual evidence for this conclusion.

In summary, the developmental trajectories of physical fitness test items exhibited significant and complex heterogeneity across different BMI groups, and the pattern of this influence varied by indicator type. Gravity-dependent indicators generally showed an “obesity disadvantage” accompanied by a certain “catch-up effect”; the absolute functional indicator revealed an “fat but fit paradox”; and the flexibility indicator supported a “normal-weight advantage.” Gender not only exerted a main effect on baseline levels across all items, but also significantly moderated the between-BMI-group differences in the rate of change for most items. These findings underscore the need to consider the joint influence of BMI and gender in physical fitness assessment and intervention.

## 4 Discussion

This study employed a four-year longitudinal design and a Multi-Group Latent Growth Curve Modeling (MG-LGCM) framework to analyze the developmental trajectories of PFI and various physical fitness test items among college students. By separating the intercept (initial status) from the slope (rate of change), we found significant spatiotemporal heterogeneity driven by the intersection of BMI and gender. Notably, the data delineate a complex physiological landscape characterized by an “obesity disadvantage” in gravity-dependent indicators and relative functional indicator, a distinct “catch-up effect” in absolute strength and PFI (23), and a “fat but fit paradox” in absolute functional capacity.

### 4.1 The “obesity disadvantage” and the biomechanical catch-up effect

For gravity-dependent indicators (e.g., endurance running, pull ups (male)/1-min sit-ups(female)) and the relative functional indicator (i.e., FWI), a pronounced obesity disadvantage was evident at baseline. The markedly lower initial performance in the overweight and obese groups can be largely attributed to the biomechanical constraints of excess adiposity, which alters joint kinematics and disproportionately increases the energetic cost of mechanical work against gravity (24, 25). Overweight and obese students consistently exhibit marked deficits in weight-bearing test items compared to their normal weight peers (26). Furthermore, the secular decline in physical fitness among Chinese college students over the past decade has been closely coupled with rising obesity rates (4).

The longitudinal MG-LGCM analysis, however, uncovered a highly novel catch-up effect: higher-BMI groups exhibited significantly steeper rates of improvement in strength-endurance test items compared with their normal weight peers. This counterintuitive acceleration can be explained by chronic physiological adaptation. Excess body mass functions as a continuous, albeit involuntary, form of resistance training. Foundational biomechanical research confirms that adiposity-induced mechanical overload chronically stimulates skeletal muscle architecture, augmenting the absolute muscle mass of the lower extremities and core (27). Consequently, although excess fat mass restricts initial performance, the underlying musculature adapts at an accelerated rate, enabling overweight and obese late-adolescents to narrow the performance gap in absolute force-generation test items across the college years.

### 4.2 The forced vital capacity “paradox”

A striking deviation from conventional normal-weight assumptions emerged for the absolute functional indicator. Overweight and obese students exhibited a significant baseline advantage in forced vital capacity and maintained this superiority throughout the 4-year observation period. Rather than an anomaly, this obesity paradox is firmly rooted in the principles of allometric scaling and respiratory physiology.

In young, relatively healthy populations, mild-to-moderate obesity is frequently accompanied by proportional increases in fat-free mass—including the structural dimensions of the thoracic cavity and the absolute strength of the respiratory musculature (28). Accordingly, individuals with higher BMI often record larger absolute lung volumes (29). Recent epidemiological studies on Chinese college students echo this phenomenon, noting that while absolute forced vital capacity often correlates positively with BMI, creating a “fat but fit” illusion, this advantage disappears when normalized to body weight (13). This highlights the ongoing debate surrounding the “fat but fit paradox” in functional fitness (30). Elevated absolute respiratory volumes in heavier individuals do not necessarily translate to superior metabolic or cardiorespiratory health (30). These findings must be interpreted with caution, however. Under the current Chinese physical fitness test, absolute forced vital capacity accounts for 15% of the total score. Consequently, overweight and obese students may achieve higher scores in this component, but this grading advantage does not equate to genuinely superior pulmonary function. Although the results accurately reflect the raw values stipulated by standardized physical fitness testing protocols, absolute forced vital capacity does not account for the metabolic demands of the accompanying adipose tissue. This paradox may therefore mask a decline in relative cardiopulmonary efficiency, underscoring a critical limitation of relying solely on absolute physiological benchmarks, as emphasized in contemporary global health guidelines (31).

### 4.3 Biomechanical restrictions and the normal-weight advantage in flexibility

In contrast to the dynamic catch-up observed in strength test items, the sit-and-reach test demonstrated a persistent normal-weight advantage. Normal weight students maintained superior flexibility from freshman to senior year. This trajectory is primarily governed by mechanical interference rather than muscular adaptation. Subcutaneous and visceral adipose tissue around the abdomen and thighs physically obstructs pelvic rotation and hip flexion during the trunk-forward reaching motion (32). Unlike muscular strength, which can adapt to chronic loading, joint range of motion remains mechanically restricted by tissue mass, corroborating evidence that higher BMI is directly associated with diminished musculoskeletal flexibility (33).

### 4.4 Gender dimorphism and the COVID-19 period effect

The analysis identified a pervasive gender dimorphism, characterized not merely by baseline differences but by divergent developmental slopes. In explosive and gravity-dependent indicators, the performance gap between males and females widened significantly over time. Crucially, interpreting these trajectories requires contextualizing the specific timeline of the students in our sample, which coincided directly with the COVID-19 pandemic and the subsequent recovery phase.

Biologically, males aged 18–22 continue to benefit from the late stages of testosterone-driven anabolism, facilitating ongoing accretion of muscle mass, whereas females generally reach hormonal stabilization earlier (34). Behaviorally, however, the pandemic exerted a profound, gender-specific impact on physical activity. Longitudinal surveillance confirms that the COVID-19 pandemic exerted a profound and lasting detrimental impact on the physical fitness of college students (2). Recent systematic reviews demonstrate that university students globally experienced a precipitous decline in physical activity during the 2020-2021 lockdowns (35, 36). However, recent evidence indicates a distinct “rebound effect” post-pandemic, where strength-related performance recovered more rapidly than cardiovascular endurance (37). As campus restrictions were lifted after 2021, the steeper slopes observed in male trajectories likely reflect a stronger rebound effect in vigorous, resistance-based sports, which males historically engage in at higher rates than females (23). Conversely, females maintained their baseline advantage in flexibility with no significant slope degradation, suggesting a resilient preference for home-based or low-intensity mobility exercises that were less disrupted by pandemic closures (38, 39).

### 4.5 Methodological strengths and practical implications

A defining strength of the present study lies in its rigorous methodological framework. The shift from cross-sectional snapshots to a longitudinal MG-LGCM paradigm represents a critical advance in sports science (6, 40). By mathematically disentangling initial status from unobserved developmental trajectories, this study provides a robust spatiotemporal map of physical fitness evolution across a major global disruption. These findings challenge the monolithic categorization of obesity in physical education, advocating for targeted, trajectory-based interventions that account for the unique biomechanical catch-up effects and shifting behavioral patterns of different BMI and gender groups. However, to further refine such interventions, future research must address current limitations by incorporating precise body composition measurements (rather than relying solely on BMI) and accounting for critical unmeasured covariates, including dynamic physical activity levels, dietary factors, and socioeconomic status.

## 5 Conclusions

This study employed multi-group latent growth curve modeling to characterize the four-year developmental trajectories of physical fitness among college students stratified by BMI category. The results reveal pronounced heterogeneity that challenges monolithic assumptions about weight and fitness. Overweight and obese students exhibited an initial “obesity disadvantage” in gravity-dependent indicators, yet they demonstrated a robust biomechanical “catch-up effect,” with significantly steeper rates of improvement in strength-endurance and explosive power. For absolute functional capacity, a clear “fat but fit paradox” emerged: higher-BMI groups entered college with superior forced vital capacity and maintained this advantage throughout the study period. By contrast, flexibility displayed a persistent normal-weight advantage, suggesting that excess adiposity may mechanically constrain range of motion irrespective of training adaptations. Gender consistently moderated both initial status and rates of change, with males generally accelerating faster in most motor domains while females preserved their baseline superiority in flexibility. Contextualized within the post-pandemic recovery phase, these divergent trajectories highlight the limitations of applying uniform, weight-centric fitness benchmarks, suggesting a shift toward more tailored, trajectory-based evaluation frameworks in collegiate physical education and health promotion.

Several limitations should be acknowledged. First, the sample was drawn from a single provincial student population during the COVID-19 recovery phase; while this provides valuable contextual insights, replication across broader populations is warranted to confirm generalizability. Second, while our methodology robustly mapped developmental trajectories using standard physical fitness metrics, it did not incorporate granular covariates such as precise body composition, physical activity levels, dietary habits, and socioeconomic status. Future longitudinal research should integrate these multidimensional variables and extend the observation window beyond graduation to further elucidate the underlying mechanisms driving the observed paradoxes. Despite these scope boundaries, this investigation provides robust longitudinal evidence that physical fitness development in emerging adulthood is a dimension-specific, non-uniform process jointly sculpted by BMI and gender, offering a nuanced foundation for designing targeted intervention strategies.

## Data Availability

The data analyzed in this study is subject to the following licenses/restrictions: the dataset analyzed in this study is not publicly available due to strict privacy, ethical, and legal restrictions regarding personal health information. The data is managed by the Sichuan Province Big Data Research and Joint Application Technology Center of Student Health, and our data use agreement explicitly prohibits the public dissemination of this dataset to ensure student anonymity and data security. However, the de-identified datasets used during the current study are available from the corresponding author upon reasonable academic request, provided that the requesting party obtains the necessary ethical clearance and permission from the data governing authority. Requests to access these datasets should be directed to Zhou He, zhou.he@swjtu.edu.cn.
